# The Evolutionary Theory of Lipedema: A Perspective on Energy Storage and Chronic Inflammation

**DOI:** 10.7759/cureus.88809

**Published:** 2025-07-26

**Authors:** Alexandre C Amato

**Affiliations:** 1 Vascular Surgery, Amato Instituto de Medicina Avançada, São Paulo, BRA

**Keywords:** adhd, attention deficit hyperactivity disorder (adhd), lipedema, obesity, obesity and diabetes, theory

## Abstract

Lipedema, characterized by the disproportionate accumulation of fat in the lower extremities, pain, and tenderness, is frequently misdiagnosed and underestimated. This evolutionary perspective reframes lipedema as an ancestral adaptive mechanism for energy storage, crucial in prehistory for female survival during periods of food scarcity. Subcutaneous fat, predominant in lipedema, conferred energetic, thermoregulatory, and cardioprotective advantages - especially for women during pregnancy and lactation - unlike men’s visceral fat, which is geared toward rapid mobilization. In the modern context, inflammatory triggers such as pollution, stress, and gluten-rich diets exacerbate chronic inflammation, turning lipedema into a clinical challenge. Inflammation acts as a warning signal, and its management - rather than indiscriminate surgical removal of adipose tissue, an essential endocrine organ - is fundamental. Extensive liposuction may lead to postoperative adipose endocrine insufficiency, with metabolic and hormonal imbalances. Conservative strategies, such as ketogenic or gluten-free diets, moderate exercise, and stress management, foster a favorable metabolic environment, allowing mobilization of stored fat.

This editorial advocates for a holistic and individualized approach, highlighting the connection between lipedema and conditions such as attention-deficit/hyperactivity disorder (ADHD), calls for further research into the interaction between genetics, inflammation, and environmental factors, and urges increased awareness of lipedema as a legitimate medical condition.

## Editorial

Lipedema is a chronic condition marked by the disproportionate accumulation of fat in the lower extremities, often accompanied by pain, tenderness, and easy bruising. Despite an estimated prevalence of 12.3% among adult women in Brazil, delayed diagnosis and confusion with obesity or lymphedema remain common [1,2]. In this evolutionary perspective, I propose that lipedema is not merely a dysfunction, but an ancestral adaptive mechanism that becomes maladaptive in a modern context of food abundance and inflammatory triggers.

In prehistory, survival depended on the ability to store energy for times of scarcity. Fat, yielding 9 calories per gram, is a more efficient energy reservoir than carbohydrates or proteins (4 calories per gram each) [3]. Subcutaneous adipose tissue, predominant in lipedema, served as a vital energy reserve, offering thermal protection and metabolic support, especially for women during pregnancy and lactation. The gynoid distribution of fat - concentrated in the thighs, hips, and legs - stored energy efficiently and provided climatic and cardiovascular protection, in contrast to visceral fat, more common in men [4-6].

In prehistoric societies, men and women played distinct roles that shaped their energy storage metabolisms. Women, often responsible for gestation, lactation, and childcare, developed a metabolism geared toward long-term energy conservation, storing subcutaneous fat to withstand prolonged food shortages or elevated demands such as pregnancy. While not all fat accumulation is adaptive - particularly in contemporary contexts of abundance - this hypothesis posits that lipedema may stem from an ancestral mechanism that prioritized long-term energy conservation in women, becoming problematic only under modern conditions. In this context, genes such as HLA-DQ2 and HLA-DQ8 - associated with gluten sensitivity - were not disadvantageous, since the diet aligned with a ketogenic metabolism, favoring the mobilization of stored fat [7]. Female hormones, like estrogen and progesterone, marked the transition from childhood to caregiver status. Milestones such as menarche and pregnancy signaled the body's need to ensure survival for the benefit of offspring and the community, irrespective of aesthetic concerns or symptoms. The mere shift in social role was insufficient to activate this energy-storage mechanism; the body required a signal that the environment was hostile. This metabolic warning was characterized by oxidative stress. Nature prioritizes survival over the appearance of the legs. In contrast, men - often engaged in physically intensive activities like hunting or territorial defense - evolved a metabolism optimized for rapid energy mobilization, with predominant visceral fat, which allowed immediate access to calories for short-duration efforts but carried a higher cardiovascular risk [6,8]. This metabolic difference resulted in greater female longevity, as women today live, on average, seven years longer than men [8].

In prehistoric times, the diet was predominantly ketogenic, with low carbohydrate intake and no wheat or gluten. Women predisposed to lipedema, characterized by greater subcutaneous storage, were more likely to survive famine and cold, ensuring the continuity of offspring. However, female hormones alone do not trigger fat deposition in lipedema; the body must receive a "warning" of aggression, signaling the need to store energy. That warning is inflammation, triggered by environmental factors such as water scarcity (a signal of impending food shortage), hypocaloric diets (interpreted as famine), or tribal conflicts. In such situations, the woman remained in the shelter caring for children - where muscle development was counterproductive and fat loss meant sacrificing vital energy reserves. Conversely, men expended energy rapidly in hunts or wars, reinforcing the metabolic gender difference [6,8].

Interestingly, lipedema may also be associated with conditions such as attention-deficit/hyperactivity disorder (ADHD), which studies suggest conferred an evolutionary advantage. The hyperactivity and impulsivity may have aided the exploration of new territories and rapid decision-making in hostile environments [9]. Although empirical validation in ancient populations is challenging, modern simulations and genetic studies support that ADHD traits may have provided evolutionary edges in foraging and decision-making. My research has identified a significant correlation between lipedema and ADHD symptoms, suggesting that strategies to improve clinical adherence could optimize lipedema treatment [10]. In this study of 354 women, the prevalence of ADHD symptoms was 76.9% in the lipedema group versus 54% without, yielding a relative risk of 1.424 (p < 0.0001), with stronger correlations in impulsivity domains, potentially reflecting evolutionary overlaps in adaptive traits turned maladaptive today. Addressing ADHD could thus enhance treatment compliance, such as through motivational interviewing or digital reminders.

In the modern era, the abundance of calorie-dense foods, exposure to inflammatory triggers (such as pollution, stress, and gluten), and sedentary lifestyles turn this ancestral mechanism into a burden. Inflamed lipedemic fat becomes rigid, painful, and resistant to mobilization, perpetuating a cycle of chronic inflammation [11].

Inflammation is therefore central to lipedema, functioning as an endogenous alert system [12-14]. Symptoms such as pain, tenderness, and a sensation of heaviness signal oxidative stress and chronic inflammation, akin to a headache indicating an underlying issue [15]. The "cup" metaphor illustrates the limited metabolic capacity of everyone to handle inflammatory triggers. When that capacity is exceeded, lipedema overflows into symptoms, signaling the need for intervention.

The high prevalence of HLA-DQ2 and HLA-DQ8 in lipedema patients (47.4% and 22.2%, respectively) suggests that gluten may exacerbate inflammation in genetically predisposed individuals [7]. Gluten-free or ketogenic diets - mimicking prehistoric eating - can reduce inflammation and improve symptoms, as shown in case studies reporting significant improvements with non-surgical approaches [16].

Indiscriminate surgical removal of adipose tissue, while relieving local symptoms, does not address the root cause - inflammation - and may mask underlying inflammation, leading to "post-surgical adipose endocrine insufficiency," an emerging concept describing metabolic and hormonal imbalances resulting from loss of an active endocrine organ [17,18]. Adipose tissue produces over 600 adipokines, including leptin, adiponectin, and inflammatory mediators, which regulate metabolism, insulin sensitivity, and hormonal homeostasis [19-21]. Surgical removal of adipose tissue alters a woman's metabolism, undermining her ability to regain metabolic health and trapping her in a vicious cycle [22,23]. That said, in patients with advanced functional limitations, targeted liposuction may offer palliative relief when conservative options fail, though it remains experimental and demands vigilant monitoring for metabolic sequelae. 

The evolutionary theory of lipedema suggests that management should focus on reducing inflammation and deactivating the hyperactive storage mechanism. Extreme hypocaloric diets may be counterproductive, signaling food scarcity and reinforcing fat retention [24]. My research has demonstrated that strategies such as ketogenic, gluten-free, or anti-inflammatory diets; aquatic exercise; manual lymphatic drainage; and antioxidant phytotherapy can improve symptoms and aesthetics in selected cases [16]. Ultrasound, using specific criteria such as pretibial region thickness, is a promising tool for differential diagnosis, reducing underdiagnosis [25].

The association between lipedema and ADHD underscores the need for personalized strategies that consider adherence challenges in ADHD patients [11]. The Brazilian Consensus on Lipedema highlights the importance of multidisciplinary approaches, prioritizing conservative treatments and raising awareness to combat stigma [26]. While liposuction can be effective in some cases, it should be considered adjunctive and still experimental, with rigorous medium- and long-term monitoring of endocrine-metabolic parameters, as demonstrated in my recent meta-analysis [27].

The evolutionary theory of lipedema offers a new lens through which to understand its pathophysiology, highlighting the interaction between genetics, inflammation, and environmental factors. Future studies should explore all possible inflammatory triggers, the benefits of ketogenic or gluten-free diets, and the long-term impacts of liposuction. Raising awareness of lipedema as a legitimate medical condition is crucial to reduce stigma and promote early diagnosis. Transforming lipedema from a burden into an opportunity for balanced health requires treating inflammation as the root cause, while preserving the evolutionary advantages of female metabolism [2,28].

Conclusion

Lipedema, viewed through an evolutionary lens, reveals a complex interplay of ancestral survival mechanisms and modern environmental challenges. Before resorting to surgical interventions like liposuction, which may disrupt the endocrine functions of adipose tissue, we must deepen our understanding of lipedema’s inflammatory roots and long-term metabolic consequences. A cautious, multidisciplinary approach - emphasizing inflammation reduction through diet, lifestyle, and conservative therapies - offers a path to manage symptoms while preserving the body’s adaptive capabilities. Comprehensive research and awareness are essential to ensure treatments that honor the intricate balance of female metabolism, avoiding irreversible interventions with uncertain long-term impacts.
